# Cyclic AMP Enhances TGFβ Responses of Breast Cancer Cells by Upregulating TGFβ Receptor I Expression

**DOI:** 10.1371/journal.pone.0054261

**Published:** 2013-01-18

**Authors:** Ilka Oerlecke, Elke Bauer, Angela Dittmer, Benjamin Leyh, Jürgen Dittmer

**Affiliations:** Klinik für Gynäkologie, Universität Halle, Halle/Saale, Germany; Wayne State University School of Medicine, United States of America

## Abstract

Cellular functions are regulated by complex networks of many different signaling pathways. The TGFβ and cAMP pathways are of particular importance in tumor progression. We analyzed the cross-talk between these pathways in breast cancer cells in 2D and 3D cultures. We found that cAMP potentiated TGFβ-dependent gene expression by enhancing Smad3 phosphorylation. Higher levels of total Smad3, as observed in 3D-cultured cells, blocked this effect. Two Smad3 regulating proteins, YAP (Yes-associated protein) and TβRI (TGFβ receptor 1), were responsive to cAMP. While YAP had little effect on TGFβ-dependent expression and Smad3 phosphorylation, a constitutively active form of TβRI mimicked the cAMP effect on TGFβ signaling. In 3D-cultured cells, which show much higher levels of TβRI and cAMP, TβRI was unresponsive to cAMP. Upregulation of TβRI expression by cAMP was dependent on transcription. A proximal TβRI promoter fragment was moderately, but significantly activated by cAMP suggesting that cAMP increases TβRI expression at least partially by activating TβRI transcription. Neither the cAMP-responsive element binding protein (CREB) nor the TβRI-regulating transcription factor Six1 was required for the cAMP effect. An inhibitor of histone deacetylases alone or together with cAMP increased TβRI expression by a similar extent as cAMP alone suggesting that cAMP may exert its effect by interfering with histone acetylation. Along with an additive stimulatory effect of cAMP and TGFβ on p21 expression an additive inhibitory effect of these agents on proliferation was observed. Finally, we show that mesenchymal stem cells that interact with breast cancer cells can simultaneously activate the cAMP and TGFβ pathways. In summary, these data suggest that combined effects of cAMP and TGFβ, as e.g. induced by mesenchymal stem cells, involve the upregulation of TβRI expression on the transcriptional level, likely due to changes in histone acetylation. As a consequence, cancer cell functions such as proliferation are affected.

## Introduction

The TGFβ signaling pathway is fundamentally involved in cancer progression [1], [2]. Classically, by interacting with the TGFβ receptor II (TβRII) TGFβ triggers the interaction between TβRII and TβRI, which leads to the activation of the TβRI kinase [3]. As a consequence, a downstream target of TβRI, such as Smad3, is phosphorylated inducing its translocation to the nucleus where it together with Smad4 drives the expression of plethora of genes including genes involved in proliferation, invasion and metastasis [4]. Besides the canonical pathway, TGFβ has been reported to interfere with the activity of other proteins and signaling pathways, such as the Ras/Raf/MEK1/ERK1/2 pathway or PAR6 [5]. TGFβ function in cancer is ambivalent in nature. In early stages of cancer, it acts as a tumor suppressor by inhibiting proliferation, whereas, in later stages, it promotes cancer progression, e.g. by triggering epithelial-mesenchymal transition, an important step towards metastasis [6], [7].

Another important pathway in cancer progression is the cAMP/protein kinase A (PKA) signaling cascade. cAMP is produced by adenylate cyclases in response to the activation of G protein-coupled receptors (GPCRs) [8]. cAMP activates PKA which, in turn, phosphorylates certain transcription factors, such as CREB or activating transcription factor-1 (ATF-1) [9]. A genome-wide study revealed that more that 4000 promoters are occupied by phosphorylated CREB suggesting CREB plays an important general role in transcriptional control [10]. CREB has been reported to promote proliferation, migration, invasion and bone metastasis of breast cancer cells [11]. In addition, higher expression of CREB has been shown to correlate with poorer prognosis in breast cancer [12]. PKA plays a role in development of resistance of breast cancer cells to the anti-estrogen tamoxifen [13].

Given the importance of the TGFβ and cAMP pathways, we explored the possibility that these pathways cross-talk in breast cancer cells. In vivo breast cancer cells can either be attached to a substratum, e.g. invasive breast cancer cells to matrix proteins, or tethered to each other, e.g. cells in ductal carcinoma in situ or in pleural effusions. We therefore performed our studies in conventional 2D adhesion cultures and in 3D suspension cultures. We found that, in 2D cultures, a rise in the cAMP level led to enhanced TGFβ responses of a number of important cancer-related genes. This was accompanied by an increase in Smad3 phosphorylation and by an upregulation of the expression of the TGFβ receptor I. However, in 3D-cultured cells, where basal cAMP and Smad3 levels were found to be significantly higher, TGFβ responses were also higher and could not be further increased by stimulating cAMP production. These data suggest an involvement of the cAMP pathway in TGFβ-regulated gene expression in breast cancer.

## Materials and Methods

### Cell Lines and Plasmids

MDA-MB-231 cells were obtained from T. Guise [14] and their identity was confirmed by an authentication 16 Loci analysis (LGC standards). The cells were maintained in RPMI medium supplemented with 10% fetal calf serum (FCS, Pan Biotech) in the absence of antibiotics. hMSCs were isolated and propagated as described [15]. For 3D cultures, cells were grown as previously described [16]. Briefly, after trypsinization five million cells were grown on top of a layer of 2% Seakem GTG agarose (dissolved in PBS) without the addition of matrix proteins. The freely floating cells quickly aggregated to form aggregates (Figure S1A). Promoter assays were performed with either 3TP-luc containing the TGFβ-responsive element between −636 and −740 of the human PAI-1 promoter [17] or TβRI(-392/+21)/pGL4. For TβRI(-392/+21)/pGL4 synthesis, the TβRI-specific sequence from −392 and +21 was PCR-amplified from genomic DNA by using Pfu polymerase (Fermentas) and inserted into the EcoRV site located in the multiple cloning site of the pGL4 vector (Promega). Correct sequence and positioning of the TβRI promoter fragment within the pGL4 vector were confirmed by DNA sequencing (MWG Eurofins). For overexpression of Smad3, Six1 or a constitutively active form of TβRI, pEXL-Flag-Smad3 [18], Six1FL [19] or pcDNA3/TβRI(T204D) [20], respectively, were used.

### Activators, Inhibitors and Antibodies

Where indicated, 10 µM forskolin (dissolved in DMSO; Calbiochem) and/or 10 ng/ml recombinant human TGFβ1 (dissolved in 1 mg/ml bovine serum albumin in 4 mM HCl; R&D) were added. Actinomycin D (Calbiochem) was dissolved in 50% DMSO and used at a final concentration of 5 µg/ml. For the inhibiton of TβRI or HDACs, cells were treated with 10 µM LY364947 (Tocris Bioscience) or 2 µM HDAC inhibitor III (Calbiochem), respectively. Following antibodies were used for Western blot analysis. In brackets final dilution, expected apparent molecular weight(s) of the recognized protein(s) and provider are given. Rabbit polyclonal antibodies anti-ERK1/2 (1∶2000, ∼42 and ∼44 kD, Cell Signaling Technology), anti-Smad2/3 (1∶1000, 55–60 kD, Santa Cruz), anti-P- Ser423/S425-Smad3 (1∶1000, 55–60 kD, R&D), anti-TIMP-1 (1∶1000, ^∼^29 kD, GeneTex), anti-TGFβ receptor I (1∶500, ∼52 kD, Cell Signaling Technology), anti-YAP and anti-phospho-Ser127-YAP (1∶1000, 65–75 kD, both Cell Signaling Technology), mouse monoclonal anti-Cox-2 (1∶500, ∼72 kD, DakoCytomation, clone CX-294,), anti-GAPDH (1∶10000, ∼36 kD, Ambion) and anti-PAI-1 (1∶1000, American Diagnostica) and rabbit monoclonal antibodies anti-CREB and anti-pS133-CREB (1∶1000, ∼43 kD, Epitomics). As secondary antibodies anti-rabbit or anti-mouse peroxidase conjugates (1∶2000, Cell Signaling Technology) were used.

### Quantitative RT-PCR

RNA isolation, cDNA synthesis, and quantitative PCR (Q-PCR) were carried out as described [21] with some modifications. Primers for Q-PCR were purchased from Eurofins MWG and are listed in Table 1. Briefly, total RNA was isolated by using Nucleospin RNA II (Macherey & Nagel) according to the manufacturer’s protocol. For cDNA synthesis, 1 µg of total RNA was mixed with 1 µl of 10 mM dNTPs (Eppendorf), 1 µl of RNasin (Promega), and 1 µl (100 ng) of random hexamers (Amersham Biosciences) in a total volume of 13 µl and incubated at 65°C for 5 min and quickly cooled on ice. After addition of 4 µl of 5× strand buffer and 2 µl of 0.1 M DTT, the primers were allowed to anneal to the RNA at 25°C for 2 min. For cDNA synthesis, 1 µl of Superscript II (200 units/µl; Invitrogen) was added and the mixture incubated at 25°C for 10 min and then at 42°C for 50 min. The reaction was stopped by keeping the mixture at 70°C for 15 min. For Q-PCR in a Bio-Rad iCycler, 10 µl of ABsolute QPCR SYBR Green Fluorescein mix (ABgene) was mixed with 1.25 µl of each primer (2.5 pmol), 2 µl of cDNA (1∶20 diluted), and 5.5 µl of water. After activation of the polymerase at 95°C for 15 min, 40 PCR cycles were run. In each cycle, DNA was denatured at 95°C for 15 s, followed by primer annealing at 60°C for 1 min and DNA synthesis at 72°C for 1 min. Each sample was analyzed in duplicate. The results were analyzed by using the iQ5 Optical System software version 2.0 (Bio-Rad). Relative RNA levels of genes were calculated by the comparative *Ct* (2^−ΔΔ*Ct*^) method. For normalization, GAPDH and HPRT genes were used.

**Table 1:** Primers for Q-PCR.

Gene	Forward primer (5′->3′)	Reverse primer (5′->3′)
Cox-2	GCAAATTGCTGGCAGGGTT	TCTGTACTGCGGGTGGAACAT
GAPDH	GAAGGTGAAGGTCGGAGT	GAAGATGGTGATGGGATTTC
CREB	GCTGCCTCTGGAGACGTACAA	GCTAGTGGGTGCTGTGCGA
HPRT	GGACAGGACTGAACGTCTTGC	TGAGCACACAGAGGGCTACAA
MMP**-**9	CCCGGACCAAGGATACAGTTT	GGAATGATCTAAGCCCAGCG
MMP-10	TGGAGCAAGGCTTCCCCTAGA	TGATGACTTTCCAGGAGTTGAGC
p21	CTGTGATGCGCTAATGGCG	CGGTGACAAAGTCGAAGTTCC
PAI-1	GGCCATGGAACAAGGATGAGA	GACCAGCTTCAGATCCCGCT
PTHrP	ACCTCGGAGGTGTCCCCTAAC	TCAGACCCAAATCGGACGG
Six1	TGCTTCAAGGAGAAGTCGAGG	GGATTGTGCGCGTACCACT
Smad3	GTGGATGGCTTCACCGACC	TTGACATTGGAGAGCAGCCC
TGFα	AGCCTTTTGTGGGCCTTC	GAATAACCCCAAGCAGACGG
TGFβ1	TTAGCGCCCACTGCTCCT	GAACCCGTTGATGTCCACTTG
TGFβ2	TGGCTTCACCATAAAGACAGGA	TACAAAAGTGCAGCAGGGACA
TGFβ3	CTTCGTCCTCAGGGTTGCC	CTGCGAGAGCTTCAGGACTTC
TIMP-1	CTGTTGTTGCTGTGGCTGAT	TGGATAAACAGGGAAACACT
TβRI	CATTGCTGGACCAGTGTGCT	CAGTGCGGTTGTGGCAGAT
TβRII	AGAAGCTGAGTTCAACCTGGGA	TGATGGCACAGTGCTCGC

### RNA Interference

Small interfering RNAs (Table 2) were purchased from Eurofins MWG. Cells were transfected by electroporation as described [15]. Briefly, cells were trypsinized, washed once with RPMI medium (without serum), and resuspended in RPMI medium at a density of ∼8 million cells per ml. For each transfection, 250 µl of the cell suspension was mixed with 5 µl of a siRNA (500 pmol) stock solution in water and electroporated by using a Bio-Rad GenePulserX-Cell (250 V, 800 µF). After incubation on ice for 30 min, cells were mixed with growth medium and seeded into cell culture dishes (Nunc). Cells were grown for 2–3 days before starting treatment with forskolin or TGFβ1 for 3–24 h as indicated.

**Table 2:** List of siRNAs.

siRNA	Sense strand (5′->3′)
siLuc	CUUACGCUGAGUACUUCGA
siCREB	UGACUUAUCUUCUGAUGCA
siSix1	CCAACUCUCUCCUCUGGAA
siSmad3	CCAGUGACCACCAGAUGAA
siYAP	GACAUCUUCUGGUCAGAGA

### Protein Extraction

Plastic-attached cells in 2D cultures were washed once with PBS, scraped off the plate and harvested by centrifugation. Nuclear protein extraction was performed as described [21]. Briefly, after harvest, cells were resuspended in 400 µl of buffer A (10 mM HEPES (pH 7.9), 10 mM KCl, 0.1 mM EDTA, 0.1 mM EGTA) and placed on ice for 15 min. After addition of 50 µl 5% NP-40, the cell suspension was vortexed for 10 s and centrifuged for 30 s in a microcentrifuge at full speed. The pellet was mixed with 60 µl of buffer C (20 mM HEPES (pH 7.9), 400 mM NaCl, 1 mM EDTA, 1 mM EGTA, 1 mM DTT) and incubated on ice for 10 min. After centrifugation for 10 min, the supernatant was collected and stored at −80°C. Extraction of plasma membrane proteins was carried out as described [15]. Briefly, cells were resuspended in buffer A as described above and homogenized by five passes through a 20-gauge needle. The supernatant was stepwise centrifuged at 3000 (600 *g*) and 6500 rpm (3500 *g*) and then full speed in a microcentrifuge for 10 min each. The pellets after the first two centrifugations were discarded. The pellet of the last centrifugation contains the plasma membrane proteins and was dissolved in buffer D (5 mM HEPES (pH 7.9), 0.5 mM K-EDTA (pH 7.2), 1 mM DTT). To check for contamination of nuclear extracts by cytosolic proteins and *vice versa*, we compared the levels of the cytosolic phospho-YAP and the nuclear phospho-Smad3 in nuclear and cytoplasmic extracts by Western blot analysis. The data show very little to no cross-contamination (Figure S2).

### Western Blot Analysis

Western blot analysis was performed as described [21]. Briefly, for protein gel electrophoresis, 5–10 µg of protein (protein extract) or 40 µl (conditioned medium) were separated in a 10% SDS polyacrylamide gel and transferred to a PVDF membrane (Millipore). The membrane was blocked with 2% milk (Applichem) in washing buffer (10 mM Tris/HCl (pH 7.5), 100 mM NaCl, 1 mM EDTA) at RT for 10 min, incubated with the primary antibody in washing buffer containing 0.2% milk at RT for 1 h and washed three times in washing buffer containing 0.05% Tween 20 for 5 min each. Incubation with the secondary antibody and washing were performed as described for the primary antibody, except that the washing time was extended to 20 min each cycle. Chemiluminescent visualization of the bands was performed by using Amersham ECLPlus (GE Healthcare) followed by exposure to Hyperfilm ECL (GE Healthcare).

### Promoter Assays

For firefly luciferase-based promoter assays, 5 µg of either 3TP-luc or of TβRI(-392/+21)/pGL4.10 were transfected into the cells by electroporation as described under “RNA interference”. Where indicated, the promoter construct was co-transfected together with 5 µg of expression plasmid. Luciferase data obtained by transfection with 3TP-luc were normalized to the amount of total protein, those obtained by TβRI(-392/+21)/pGL4.10 against the renilla luciferase activity as generated by co-transfecting cells with the pGL4.74-plasmid (2.5 µg). Electroporated cells were seeded into 12-well plates. After incubation overnight, medium was replaced by fresh medium supplemented with either forskolin, TGFβ1 or both or by mock-supplemented medium and cells were incubated for additional 24 h. Cells were washed with PBS and lysed in 250 µl PLB (Promega dual luciferase reporter assay) for 15 min at RT. After clearing the lysate by centrifugation, 10 µl of each supernatant was mixed with 50 µl of Luciferase Assay Reagent II and analyzed for luciferase activity (Sirius Luminometer, Berthold Detection Systems).

### cAMP Assay

Measurements of the intracellular cAMP levels were carried out by using a cyclic AMP Enzyme Immunoassay Kit (Cayman) by following the acetylated procedure protocol of the manufacturer. Briefly, after cells were treated with 0.1 M HCl for 20 min at RT the lysate was cleared by centrifugation and acetylated. For each measurement, 50 µl of acetylated sample were mixed with 50 µl of tracer and 50 µl of cAMP-specific rabbit antibody for 17–18 h at 4°C. After addition of Ellman′s reagent absorbance at 412 nm was measured in a Spectra Max 340 PC (Molecular Devices).

### Proliferation Assay

DNA synthesis was measured by seeding cells into 96-well plates (2500 cells per well), adding 100 µl of medium/serum supplemented with 10 µM 5-bromo-2′-deoxy-uridine (BrdU), incubating for 24 h and measuring BrdU incorporation into DNA by anti-BrdU ELISA essentially as described in the manufacturer’s protocol (BrdU labeling and detection kit III, Roche).

## Results

### cAMP Enhances TGFβ-dependent Gene Expression

**Figure 1:**
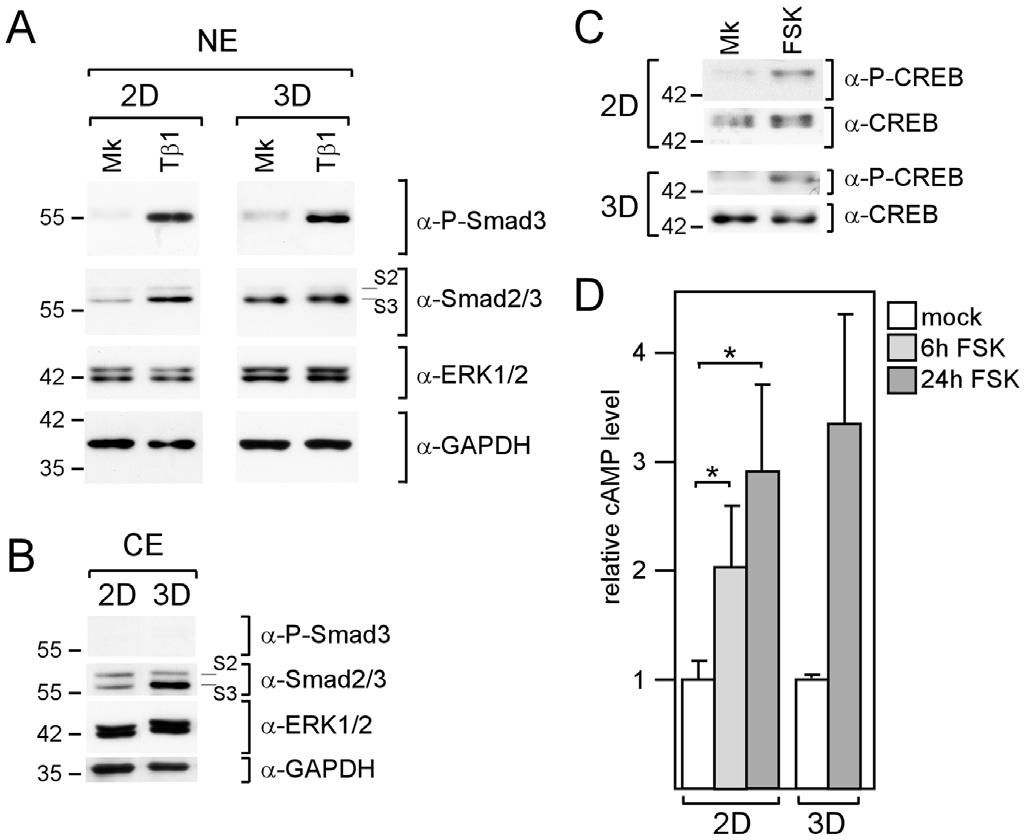
MDA-MB-231 cells are similarly responsive to forskolin and TGFβ in 2D and 3D cultures. Cells were analyzed for changes in the phosphorylation status of Smad3 (A) and CREB (B) as well as for changes in the cAMP level in response to TGFβ1 and forskolin, respectively. (A-C) Western blot analyses of nuclear extracts (NE) or cytoplasmic extracts (CE) of cells treated with mock (Mk), forskolin (FSK) or TGFβ1 (Tβ1) as indicated for 2 h to study Smad2/3 (S2/3) or CREB phosphorylation by using antibodies specific to phospho-Smad2/3 or phospho-CREB, respectively. To check for equal protein loading, blots were reprobed with anti-GAPDH and anti-ERK1/2 (A, B) or anti-CREB (C). (D) cAMP was measured by EIA as described by Material & Methods. Each bar represents the mean value of three (2D) or two (3D) independent experiments. Error bars denote S.D. * p-value <0.05 (Student’s t-test).

To study the cross-talk between the cAMP and the TGFβ signaling pathways in breast cancer cells we chose MDA-MB-231 cells, because they express functional TGFβ receptors I and II and respond to TGFβ by nuclear translocation of Smad3 and upregulation of TGFβ-responsive genes, such as PTHrP and PAI-1 [16], [20], [22]–[24]. We first analyzed Smad3 phosphorylation in 2D and 3D cultures by Western blot analysis. We found that, even without addition of TGFβ, P-Smad3 could be detected under both culture conditions (Fig. 1A). This is in line with the previous observation that MDA-MB-231 cells maintain an autocrine TGFβ loop [25]. Addition of TGFβ1 substantially increased Smad3 phosphorylation under both culture conditions (Fig. 1A). However, TGFβ1 changed total Smad3 expression differently in the nucleus of 2D- and 3D-cultured cells. In 2D-cultured cells, total nuclear Smad3 levels increased along with the P-Smad3 levels. This is in agreement with the notion that the majority of nuclear Smad3 proteins is phosphorylated. In contrast, in 3D-cultured cells, no significant change in total nuclear Smad3 level could be observed in response to TGFβ1 suggesting that most of the nuclear Smad3 in these cells was not phosphorylated. Higher total Smad3 protein levels were also found in the cytoplasm of 3D-cultured cells (Fig. 1B) indicating that total cellular Smad3 protein levels were upregulated in 3D- as compared to 2D-cultured cells.

We next examined the effect of forskolin on intracellular cAMP levels and on the phosphorylation status of the cAMP effector CREB by EIA and Western blot analysis, respectively. Forskolin raised CREB phosphorylation and cAMP levels similarly in 2D and 3D cultures (Fig. 1C, D).

**Figure 2:**
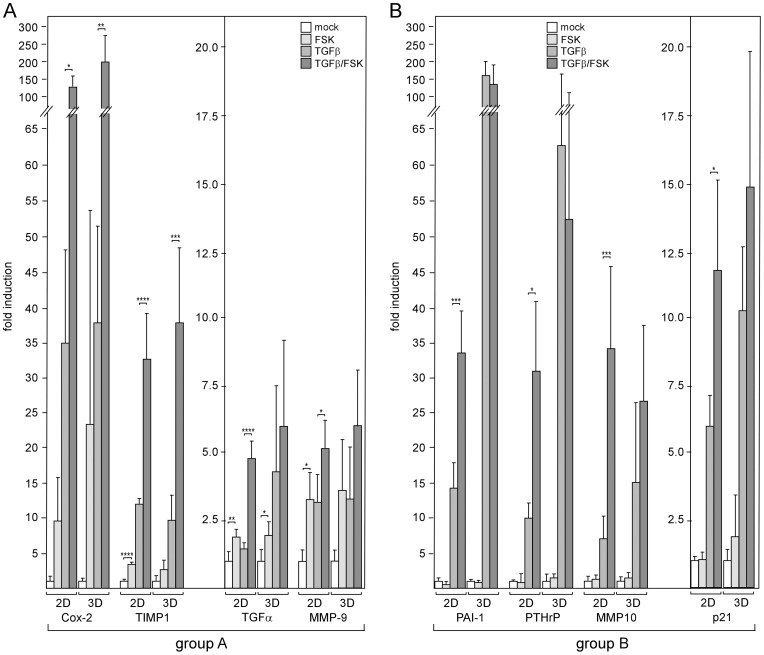
cAMP increases responses of tumor-relevant genes to TGFβ. MDA-MB-231 cells were incubated with forskolin or TGFβ1 or both or mock-treated for 24 h, lysed and analyzed for RNA levels of TIMP-1, Cox-2, PTHrP, MMP10, PAI-1, TGFα, MMP9 and p21 by Q-RT-PCR. Genes were grouped based on their abilities to respond to forskolin in the absence and presence of TGFβ (group A) or only in the presence of TGFβ (group B) in 2D-cultured cells. For graphical reasons, genes in each group were sorted by their potencies to respond to TGFβ plus forskolin. Genes showing fold induction in response to TGFβ plus forskolin >15 appear in the left graph, those displaying fold induction ≤ 15 appear in the right graph of each group. Each bar represents the mean value ± SD of 3–5 independent experiments. * p-value <0.05, ** p-value <0.01, *** p-value <0.005, **** p-value <0.001 (Student’s t-test).

**Figure 3:**
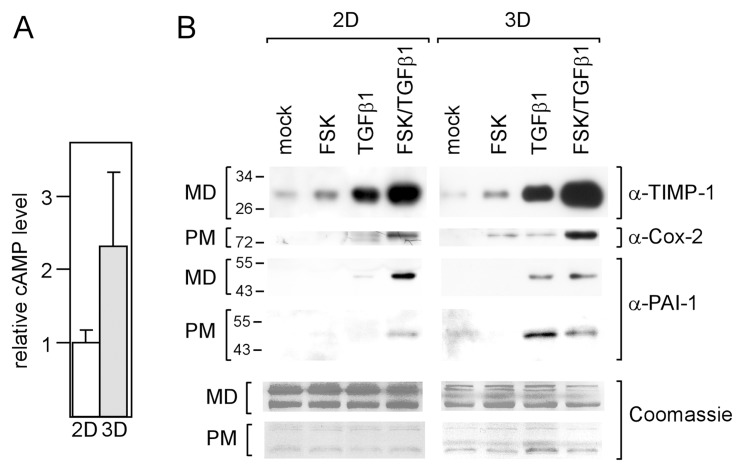
cAMP levels and forskolin effects are different in 2D - **and 3D-cultured cells.** (A) cAMP levels are higher in 3D-cultured cells compared to 2D-cultured cells. Cells were grown in 2D or 3D cultures for 24 h and analyzed for cAMP. Each bar represents the mean value ± S.D. of three independent experiments. (B) cAMP/TGFβ-induced changes in RNA levels are translated into changes in protein levels. MDA-MB-231 cells were incubated with forskolin or TGFβ1 or both or mock-treated for 24 h, lysed and analyzed for protein levels of TIMP-1, Cox-2 and PAI-1 by the Western blot technique. The level of the secretory protein TIMP-1 was measured in the medium (MD) in which cells were grown, Cox-2 protein expression was analyzed in plasma membrane (PM) extracts, PAI-1 levels were determined in both MD and PM. To check for equal loading gels were stained with Coomassie Blue.

Next, we analyzed TGFβ-responsive genes for their abilities to respond to forskolin in the presence and absence of TGFβ1. The expression patterns of eight genes (PTHrP, PAI-1, TIMP-1, transforming growth factor alpha (TGFα), matrix metalloproteases 9 (MMP9), MMP10, Cox-2 and p21) were determined by Q-RT-PCR. Two different response groups, which we termed A and B, could be distinguished. Group A genes, Cox-2, TIMP-1, TGFα and MMP9, displayed a significant increase in expression in response to forskolin under all conditions tested (Fig. 2A). This suggests that forskolin regulates these genes in a TGFβ-independent fashion. In contrast, group B genes, PAI-1, PTHrP, MMP10 and p21, showed increased expression in response to forskolin only in the presence of TGFβ1 (Fig. 2B). Given alone forskolin had no effect on PTHrP, MMP10 and p21 and decreased the expression of PAI-1. The latter effect has also been reported previously and is likely caused by a protein that regulates PAI-1 RNA stability [26]. In 3D-cultured cells, forskolin failed to enhance TGFβ-dependent expression of group B genes. In addition, PAI-1 and PTHrP showed a much stronger response to TGFβ1 under these culture conditions (Fig. 2B). This prompted us to compare the basal cAMP levels in the cells grown in 2D and 3D cultures. We found that the basal cAMP level in 3D-cultured cells was ∼2.3-fold higher than that in 2D-cultured cells (Fig. 3A). To confirm the response patterns obtained for the RNA level also for protein expression, we performed Western blot analyses for TIMP1, Cox-2 and PAI-1. Similar forskolin and TGFβ1 response patterns were observed also for the protein levels (Fig. 3B). Collectively, our data suggest that cAMP potentiates TGFβ signaling in breast cancer cells.

### cAMP Enhances the Activity of the TGFβ Effector Smad3

**Figure 4:**
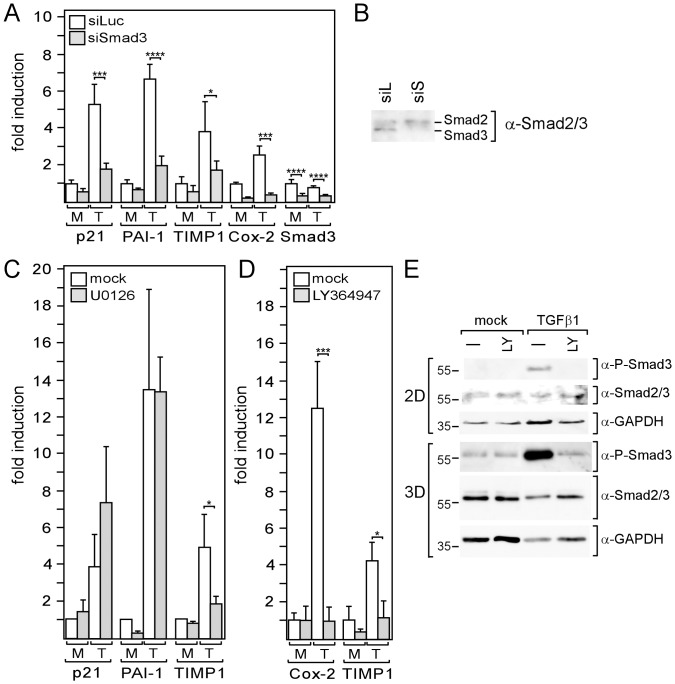
Smad3 and/or ERK1/2 are involved in the TGFβ-mediated regulation of genes in MDA-MB-231 cells. (A, B) MDA-MB-231 cells were transfected with siSmad3 or control siRNA (siLuc) and incubated for three days in 2D cultures before cells were treated with TGFβ1 (T) or mock-treated (M) for 24 h, lysed and analyzed for RNA expression of Smad3, p21, Cox-2, PAI-1 and TIMP-1 by Q-RT-PCR (A) or for nuclear Smad3 protein expression by Western blot analysis (B). (C, D, E) Cells in 2D cultures (C, D, E) or 3D cultures (E) were incubated for 24 h with TGFβ1 (T) or mock-treated (M) in the presence or absence of U0126 (C) or LY364947 (D, E) and analyzed for RNA expression of genes as indicated (C, D) or for nuclear Smad3 and phospho-Smad3 expression (E). GAPDH was used as a protein loading control (E). (A, C, D) Each bar represents the mean value ± SD of 3–6 independent experiments.* p-value <0.05, *** p-value <0.005, **** p-value <0.001 (Student’s t-test).

**Figure 5:**
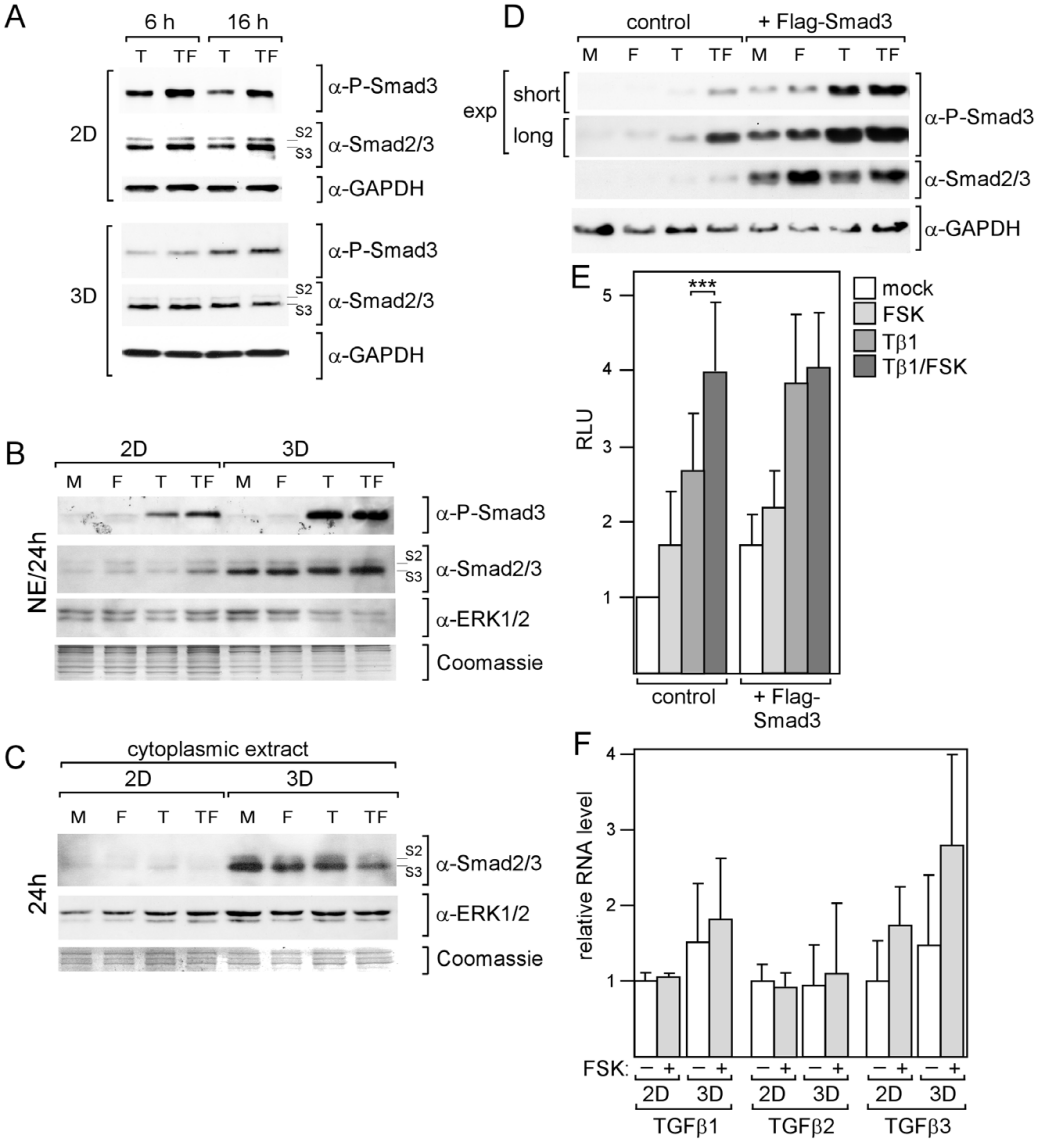
Forskolin increases phosphorylation of Smad3. (A-D) Western blot analyses of nuclear extracts (A, B, D) or cytoplasmic extracts (C) for levels of phospho-Smad3 and Smad2/3 (S2/S3). To check for protein loading the blots were reprobed with either anti-GAPDH (A, D) or anti-ERK1/2 (B, C). In B,C also Coomassie-stained proteins are shown. Cells cultured in 2D or 3D were treated with forskolin (F), TGFβ1 (T) or TGFβ1 plus forskolin (TF) or mock-treated (M) for 6, 16 or 24 h as indicated. (D) Ectopic expression of Smad3 not only leads to higher Smad3 levels but also stimulates Smad3 phosphorylation. MDA-MB-231 cells were transfected with an expression plasmid for Flag-tagged Smad3 and incubated o/n prior to treatment with forskolin (FSK), TGFβ1 (Tβ1) or TGFβ1 plus forskolin or mock-treated for 16 hours. Nuclear extracts were analyzed by the Western blot technique for the levels of phospho-Smad3, Smad2/3 and GAPDH (loading control). For P-Smad3 two different exposures (exp) of the chemiluminescent signals are shown. (E) Overexpression of Smad3 blocks the ability of cAMP to potentiate the stimulatory effect of TGFβ on the 3TP promoter containing a PAI-1 TGFβ response element. Cells were transfected with the 3TP promoter/firefly luciferase construct alone or together with a Flag-Smad3 expression plasmid and treated as indicated for 16 h. Cells were lysed and analyzed for luciferase activity. Each bar represents the mean value ± SD of nine independent experiments. *** p-value <0.005. (F) TGFβ1 RNA expression was compared in mock- and forskolin (FSK)-treated 2D- and 3D-cultured cells. Each bar represents the mean value ± SD of three independent experiments.

There are two major mechanisms by which TGFβ regulates gene expression, one involves regulatory Smads, such as Smad3, another involves ERK1/2. In both cases, TβRI mediates activation. To explore the importance of Smad3, ERK1/2 and TβRI in TGFβ-driven gene expression in MDA-MB-231 cells, we used a Smad3-specific siRNA (siSmad3), blocked ERK1/2 phosphorylation by MEK1 inhibitor U0126 and inhibited TβRI kinase activity by LY364947, respectively. Transfection with siSmad3 reduced the Smad3 RNA level by ∼3-fold (Fig. 4A), which was accompanied by a substantial decrease in Smad3 protein expression (Fig. 4B). In the presence of siSmad3, basal and TGFβ1-induced expression of p21, PAI-1, TIMP-1 and Cox-2 was downregulated by 3.3- and 5.3-fold, 1.5- and 3.3-fold, 1.8- and 2.2-fold, 4.4- and 6.3-fold, respectively (Fig. 4A). In all cases but TIMP-1, the effect of siSmad3 was considerably higher on TGFβ1-induced expression than on basal expression. This difference was most pronounced for p21 and PAI-1. In contrast, U0126 strongly interfered with TIMP-1 expression and failed to affect TGFβ-driven expression of PAI-1 and p21 (Fig. 4C). This suggests that, in MDA-MB-231 cells, TGFβ drives TIMP-1 expression through ERK1/2, whereas it primarily regulates p21, PAI-1 and Cox-2 levels through Smad3. To test the effect of LY364947, we chose Cox-2 and TIMP-1 genes as representatives for a Smad3- and for a ERK1/2-dependent TGFβ-responsive gene, respectively. In both cases, LY364947 completely abrogated the TGFβ response (Fig. 4D) indicating that TβRI is essential for both Smad3- and ERK1/2-dependent TGFβ responses. The importance of Smad3 for the TGFβ/forskolin responses of the analyzed genes prompted us to examine Smad3 phosphorylation in the presence and absence of forskolin by using Western blot analysis. As for TGFβ-driven gene expression, we found a forskolin-induced increase in Smad3 phosphorylation in 2D-cultured, but not in 3D-cultured cells (Fig. 5A, B, D). In 2D-cultured cells, forskolin also induced a slight increase in basal Smad3 phosphorylation (Fig. 5B, D) which, as mentioned above, is likely been driven by an autocrine TGFβ loop [25]. These data suggest that cAMP upregulates TGFβ-dependent gene expression by increasing TGFβ-dependent Smad3 activity. As mentioned above (Fig. 1A, B), total Smad3 levels in both nucleus (Fig. 5B) and cytoplasm (Fig. 5C) were much higher in 3D-cultured cells than in 2D-cultured cells. We explored the possibility that higher Smad3 levels may abrogate the effect of cAMP on Smad3 activity by overexpressing Smad3 in 2D-cultured cells. Overexpression of Smad3 mimicked 3D culture conditions in that it induced higher phosphorylation of Smad3 in response to TGFβ and a loss of the forskolin effect (Fig. 5D).

To analyze whether higher total levels of Smad3 also affect Smad3-driven transcription in the presence and absence of forskolin, we performed promoter assays by using the 3TP-luc construct containing the Smad3/TGFβ-responsive element of the human PAI-1 promoter between −636 and −740 [17]. In the absence of the Smad3 expression plasmid, TGFβ1 stimulated promoter activity by 2.7-fold (Fig. 5E), which could be further increased to ∼4-fold by the addition of forskolin (p<0.005, Student’s t-test). However, when Smad3 was overexpressed, forskolin failed to increase transcription and TGFβ1 alone was more effective, giving rise to a ∼4-fold induction. Hence, higher total levels of Smad3 not only led to higher Smad3 phosphorylation, but also to a higher transcriptional activity in response to TGFβ. It seems that upregulation of Smad3 levels mimicks the effect of cAMP on Smad3 phosphorylation and on Smad3-dependent transcription. We wondered, if in addition to Smad3 levels, also endogenous TGFβ levels may be different in 2D- and 3D-cultured cells. Q-RT-PCR analyses showed that, while TGFβ2 levels were equally high under both culture conditions, TGFβ1 and TGFβ3 -RNA levels in both mock- and forskolin-treated 3D-cultured cells were slightly elevated by 1.5- to 1.7-fold (Fig. 5F). These effects, however, were not statistically significant. Forskolin did not affect TGFβ1 and TGFβ2 levels, but seemed to have some effect on the TGFβ3 level, although again this was not statistically significant. In addition, TGFβ3 was expressed as much lower level than TGFβ1 and TGFβ2 (data not shown) suggesting that the weak effect of forskolin on TGFβ3 has little impact on basal Smad3 phosphorylation. Collectively these data suggest that the higher total Smad3 level in 3D-cultured cells was responsible for the higher degree of TGFβ-dependent Smad3 phosphorylation. The results further suggest that, once the Smad3 level reaches a certain threshold, cAMP has no longer an effect on Smad3 activity. Therefore, the high Smad3 levels may explain the failure of forskolin to support the TGFβ pathway in 3D-cultured cells.

### The Smad3-binding Protein YAP Down-modulates the Forskolin Effect on TGFβ-Driven Expression

**Figure 6:**
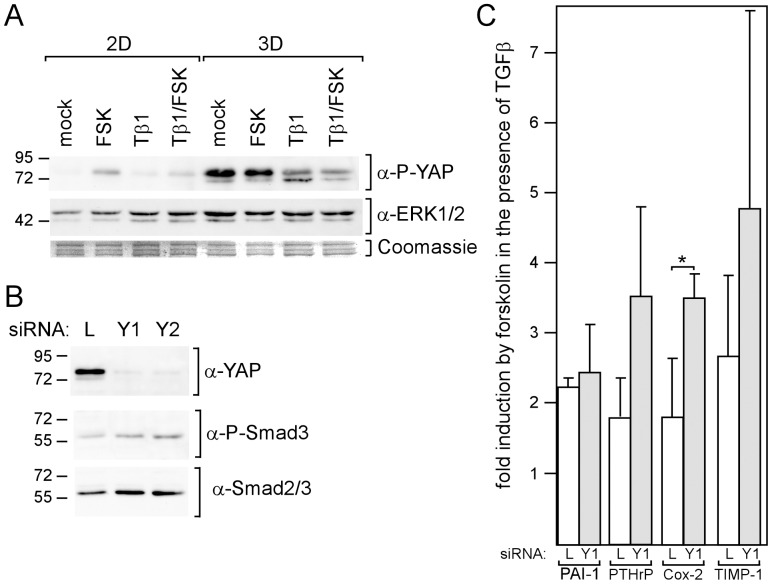
YAP does not mediate the forskolin effect on TGFβ-mediated gene expression. (A) Phosphorylation of YAP is increased by forskolin and in 3D cultures. MDA-MB-231 cells in 2D and 3D cultures were incubated with forskolin (FSK), TGFβ1 (Tβ1) or TGFβ1 plus forskolin or mock-treated o/n. Cytosolic extracts were analyzed for the phosphorylation status of YAP by the Western blot technique. To check for protein loading, the blot was reprobed with anti-ERK1/2. Also Coomassie-stained proteins are shown. (B, C) Cells were transfected with siYAP1 (Y1), siYAP2 (Y2) or siLuc (L) and incubated for three days. (B) Downregulation of YAP increases Smad3 nuclear localization. Nuclear extracts of the transfected cells were examined for YAP, phospho-Smad3 and Smad3 protein expression by Western blot analysis. (C) Downregulation of YAP increases the forskolin effect on TGFβ1-mediated gene expression. Transfected cells were incubated with forskolin (FSK) or mock-treated for 24 h and analyzed for Cox-2, TIMP-1, PTHrP and PAI-1 RNA expression by using Q-RT-PCR. Each bar represents the mean value ± SD of three independent experiments. * p-value <0.05 (Student’s t-test).

One way by which cAMP could influence Smad3 activity is by interfering with the phosphorylation of YAP. Once phosphorylated, YAP translocates from the nucleus to the cytoplasm and takes along with it Smad3, thereby reducing TGFβ/Smad3-dependent transcription [27], [28]. We found that forskolin induced YAP phosphorylation in 2D-, but not in 3D-cultured cells (Fig. 6A). However, in 3D-cultured cells, P-YAP levels were much higher. If P-YAP would inhibit Smad3 function also in MDA-MB-231 cells, lower nuclear P-Smad3 levels would be expected in the presence of forskolin and in 3D-cultured cells. However, just the opposite was the case (Fig. 5B). To further explore the role of YAP in the regulating Smad3 activity in MDA-MB-231 cells, two different YAP-specific siRNAs, siYAP1 (Y1) and siYAP2 (Y2), were used to down-regulate YAP expression (Fig. 6B). Both siRNAs had only weak effects on nuclear P-Smad3 levels (Fig. 6B). This suggests that, in MDA-MB-231 cells, YAP does not play a major role in the regulation of Smad3 activity. Nevertheless, siYAP1 seems to increase the forskolin effect on TGFβ-driven Cox-2 expression. We conclude from these experiments that, though forskolin induces YAP phosphorylation, it does not exert its enhancing effect on Smad3 phosphorylation through YAP.

### Forskolin Exerts its Effect on TGFβ-mediated Expression by Upregulating TGFβ Receptor I Expression

**Figure 7:**
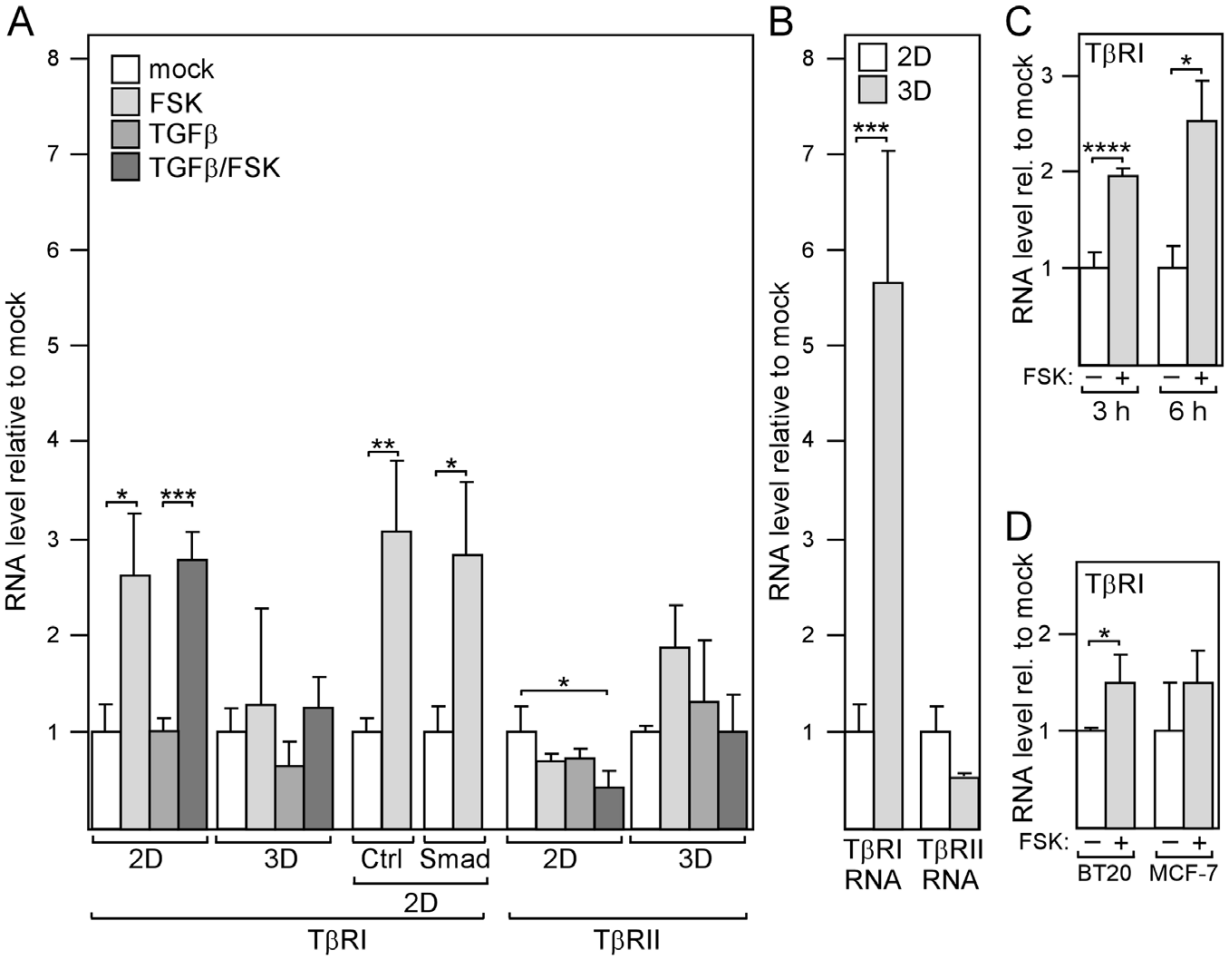
cAMP stimulates expression of TGFβ receptor I (TβRI). (A) cAMP increases the expression of TβRI, but not of TβRII. Cells were treated as indicated in 2D or 3D cultures for 24 h and analyzed for the expression of TβRI, and TβRII by Q-RT-PCR. When transfected with Flag-Smad3 (Smad) or mock-transfected (Ctrl) cells were incubated overnight before forskolin or vector (mock) was added. (B) TβRI levels are higher in 3D-cultured compared to 2D-cultured cells. Cells were grown in 2D or 3D cultures for 24 h and analyzed for TβRI-RNA and TβRII-RNA levels. Each bar represents the mean value ± S.D. of three independent experiments. (C) cAMP-dependent induction of TβRI expression is rapid. Cells were incubated with forskolin (FSK) or mock-treated in 2D cultures for 3 or 6 h before RNA expression of TβRI was analyzed by Q-RT-PCR. (D) cAMP stimulates TβRI expression also in BT-20 and MCF-7 breast cancer cells. Cells were incubated with forskolin or mock-treated for 24 h and analyzed for TβRI expression by Q-RT-PCR. Each bar represents the mean value ± S.D. of three independent experiments. * p-value <0.05, *** p-value <0.005, **** p-value <0.001 (Student’s t-test).

The protein that is critically involved in the regulation Smad3 phosphorylation in MDA-MB-231 cells is TβRI (Fig. 4E). There is evidence indicating that the expression level of TβRI is important for TGFβ signaling in breast cancer cells [19]. To explore the possibility that cAMP regulates the expression of TβRI, we analyzed the effect of forskolin on TβRI RNA levels and, for comparison, also on TβRII expression. In 2D-cultured cells where forskolin potentiated TGFβ-driven expression, forskolin also significantly increased TβRI RNA levels by ∼3-fold both in the presence and absence of TGFβ (Fig. 7A), while, in 3D-cultured cells where forskolin had no effect on TGFβ signaling, forskolin also failed to raise TβRI expression (Fig. 7A). The forskolin effects on TβRII expression showed a pattern that was different to that on TβRI expression. Forskolin-induced changes on TβRII expression could not explain the forskolin effects on TGFβ-responsive gene expression.

Interestingly, along with an elevated basal cAMP level (Fig. 3A), a 5-fold higher basal TβRI RNA level was found in 3D-cultured cells (Fig. 7B). It is therefore possible that the elevated basal cAMP level may partially be responsible for the higher basal TβRI expression in 3D-cultured cells. Since elevated Smad3 levels abrogated the forskolin effect, we wondered whether transfection with the Smad3 expression plasmid would affect the cAMP response of TβRI. However, overexpression of Smad3 did not affect the sensitivity of TβRI expression to forskolin (Fig. 7A). Hence, the loss of forskolin response of TβRI in 3D-cultured cells seems to be independent of the higher Smad3 levels in these cells. This suggests that, besides the higher Smad3 level, another event that renders TβRI unresponsive to cAMP contributes to the failure of cAMP to enhance TGFβ-driven gene expression in 3D-cultured cells. It is possible that the much higher basal TβRI expression causes 3D-cultured cells to become refractory to the potentiating forskolin effect on TGFβ-driven expression.

If cAMP exerts its effect on TGFβ-driven expression by upregulating TβRI levels, TβRI levels should rise prior to the levels of TGFβ-responsive genes, whose expression was usually examined after 24 hours. To test this, we analyzed the response of TβRI expression to forskolin also after three and six hours. As shown in Fig. 7C, a 3 h or 6 h duration of forskolin treatment was sufficient to significantly raise TβRI expression indicating that TβRI levels rose early in response to forskolin. We next wondered, if TβRI is also responsive to cAMP in other breast cancer cells. When we stimulated BT20 and MCF-7 breast cancer cells with forskolin, a ∼1.5-fold increase in TβRI RNA expression was observed (Fig. 7F) suggesting that cAMP-mediated regulation of TβRI expression is not a unique feature of MDA-MB-231 cells.

**Figure 8:**
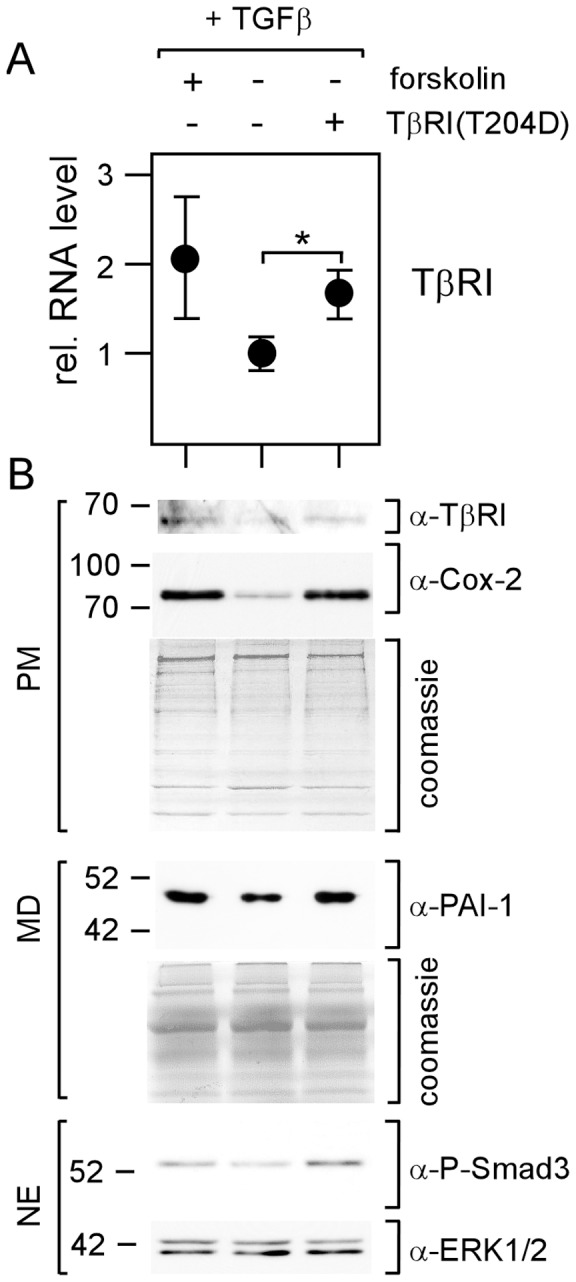
Overexpression of TβRI mimics the forskolin effect on TGFβ-driven gene expression. MDA-MB-231 cells were either transfected with 1 µg of an expression plasmid encoding a constitutively active form of TβRI, TβRI(T204D), or mock-transfected or treated with forskolin. After o/n incubation in 2D cultures cells were analyzed for TβRI RNA levels by Q-RT-PCR (A) or for protein levels of TβRI, Cox-2, TIMP-1, PAI-1, Phospho-Smad3, ERK1/2 (loading control) by the Western blot technique (B). Each circle represents the mean value ± S.D. of three independent experiments. * p-value <0.05.

To study the effect of TβRI expression on TGFβ/Smad3-mediated expression directly, we transfected MDA-MB-231 cells with TβRI(T204D), a constitutively active form of TβRI that induces Smad3 phosphorylation and has been shown to induce PTHrP expression in a TGFβ-independent manner [20]. Under conditions where the transfection with TβRI(T204D) raised TβRI expression to a level comparable to the one induced by forskolin (Fig. 8A, B), TβRI(T204D) was able to mimic the forskolin effect on Smad3 phosphorylation and on TGFβ-dependent gene expression (Fig. 8B). Collectively, these data suggest that cAMP acts on TGFβ/Smad3-mediated expression by upregulating the expression of TβRI.

### The cAMP Effect on TβRI Expression Requires Transcription, but not CREB

**Figure 9:**
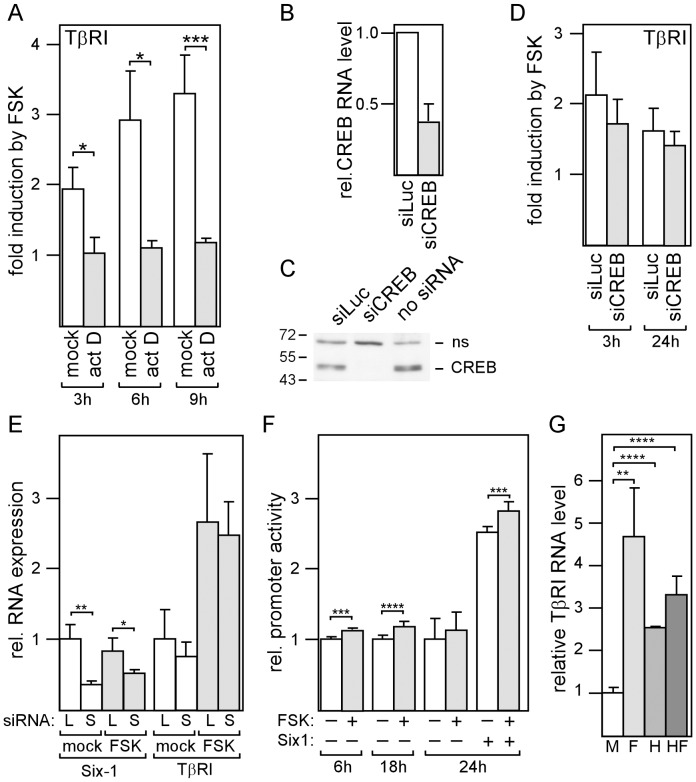
The forskolin effect on TβRI requires transcription to be active, but is independent of CREB. (A) MDA-MB-231 cells were incubated with either actinomycin to block transcription or mock-treated for 24 h and analyzed for TβRI RNA levels by Q-RT-PCR. (B-D) Cells were transfected with either siCREB, siLuc or no siRNA, incubated for three days, treated for an additional 3 or 24 h with forskolin or mock (D) and analyzed for TβRI RNA (B, D) or protein levels (C) by Q-RT-PCR or by the Western blot technique (ns = non-specific band), respectively. (E) Cells were transfected with siSix1 (S) or siLuc (L), incubated for three days, treated with forskolin or mock o/n and analyzed for Six1 and TβRI RNA expression by Q-RT-PCR. (F) The TβRI promoter is responsive to cAMP. MDA-MB-231 cells were transfected with TβRI promoter/dual luciferase construct, incubated o/n and treated with forskolin (FSK) or mock for 6, 18 or 24 hours as indicated and analyzed for firefly and renilla (control) luciferase. (G) MDA-MB-231 cells were incubated with forskolin, HDACi III, forskolin plus HDACi III or mock-treated for 24 h and analyzed for TβRI expression by Q-RT-PCR. Each bar represents the mean value ± S.D. of 3 independent experiments. Relative promoter activity denotes the ratio of firefly to renilla luciferase activity. Each bar represents the mean value ± S.D. of 3–10 independent experiments. * p-value <0.05, ** p-value <0.01, *** p-value <0.005, **** p-value <0.001 (Student’s t-test).

We next explored the possibility that cAMP regulates TβRI expression on the transcriptional level. First, we blocked transcription by treating MDA-MB-231 cells with actinomycin D. Incubation with actinomycin for 3, 6 and 9 hours completely abrogated the potentiating effect of forskolin on TβRI expression (Fig. 9A). This suggests that transcription is required for cAMP to exert its effect on TβRI expression. Since cAMP typically upregulates transcription by activating CREB, we down-regulated CREB expression by a CREB-specific siRNA (siCREB) which efficiently decreased CREB RNA and protein expression (Fig. 9B, C). However, siCREB failed to significantly down-modulate the forskolin effect on TβRI expression (Fig. 9D). The transcription factor Six1 has recently been reported to play a critical role in regulating TβRI transcription [19]. To explore the role of Six1 in cAMP-driven TβRI expression, we knocked-down Six1 expression by RNA interference. Though siSix1 substantially decreased Six1 expression, it only moderately affected TβRI expression in both forskolin- and mock-treated cells (Fig. 9E). These data suggest that neither CREB nor Six1 are required for cAMP to exert its effect on TβRI expression.

We next explored the possibility that the TβRI promoter is cAMP-responsive. We cloned the proximal TβRI promoter between nucleotides at positions −392 and +21 into the pGL4.10 vector containing the firefly luciferase gene as reporter gene. For normalization the plasmid pGL4.74, which harbors the renilla luciferase gene, was co-transfected along with the TβRI promoter plasmid. Forskolin was able to moderately but reproducibly increase TβRI promoter activity, which was statistically significant when forskolin treatment lasted 6 h or 18 h (Fig. 9F). Six1 substantially increased promoter activity as described previously [19], but failed to enhance the forskolin effect, which is in agreement with the finding that siSix1 failed to modulate TβRI expression.

cAMP has been shown to be capable of increasing histone acetylation [29], an effect which may not be seen in promoter assays. To examine whether acetylation is involved in TβRI expression in MDA-MB-231 cells, we incubated these cells with the HDAC inhibitor HDACi III. We found that HDACi III upregulated TβRI mRNA levels by ∼2.5-fold compared to a ∼4.5-fold increase by cAMP (Fig. 9G). cAMP and HDACi III together elevated TβRI levels by ∼3.2-fold. Since there was no additive effect of cAMP and HDACi III, we conclude that cAMP and HDACi III did not independently upregulate TβRI. Hence, it is possible that cAMP induces TβRI expression by interfering with histone acetylation.

### cAMP Supports the Anti-proliferative Effect of TGFβ

**Figure 10:**
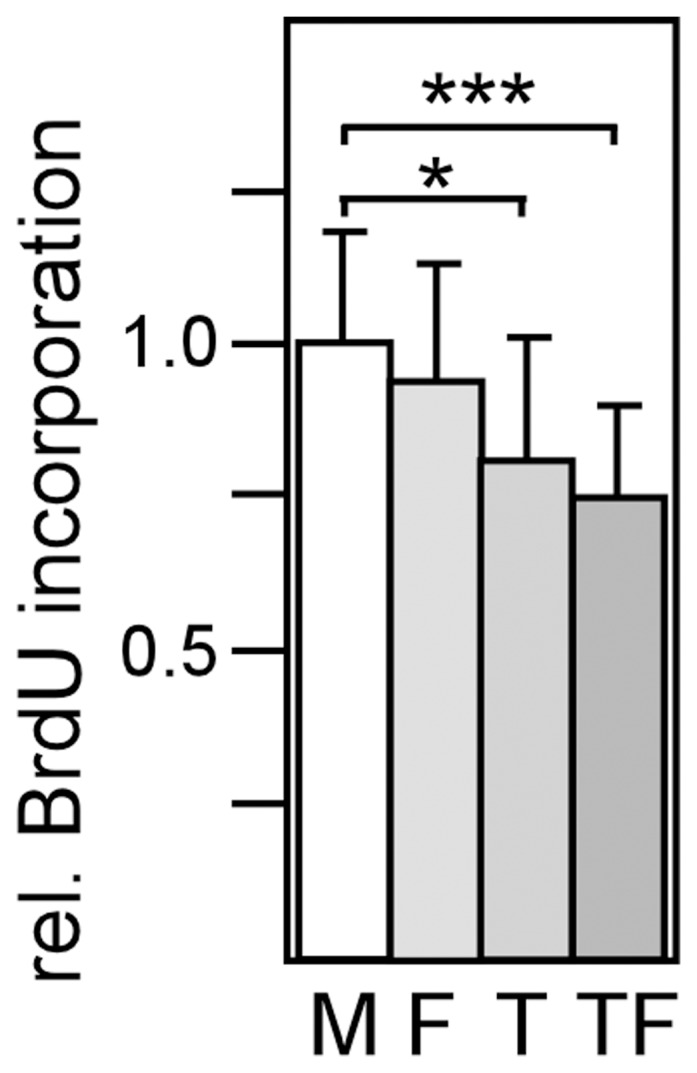
cAMP supports the anti-proliferative effect of TGF *β* **.** Incorporation of Bromo-deoxyuridine (BrdU) into DNA was measured in the presence of forskolin (F), TGFβ (T) or forskolin and TGFβ (FT) or under mock conditions (M) as described under Material and methods. Each bar represents the mean value ± S.D. of 10 independent experiments. * p-value <0.05, *** p-value <0.005, (Student’s t-test).

cAMP and TGFβ are both known to affect cellular proliferation. Since forskolin enhanced the TGFβ-dependent expression of cell cycle inhibitor protein p21, we explored the possibility that cAMP and TGFβ down-regulate proliferation in 2D-cultured MDA-MB-231 cells by measuring DNA synthesis. While forskolin alone had no significant effect on DNA synthesis, TGFβ alone reduced DNA synthesis significantly by 19% (Fig. 10). Both agents together decreased DNA synthesis further, leading to a 25% drop in proliferation compared to control conditions. This shows that, in the presence of both cAMP and TGFβ, proliferation of MDA-MB-231 is substantially reduced.

### Mesenchymal Stem Cells can Simultaneously Activate the cAMP and TGFβ Pathways in Breast Cancer Cells

**Figure 11:**
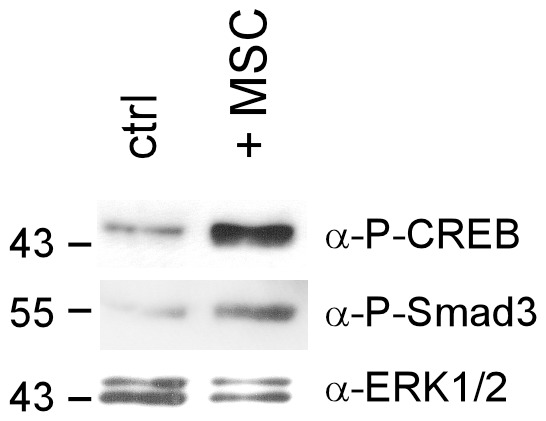
hMSCs activate the TGFβ and cAMP signaling pathway. MDA-MB-231 cells were co-cultured with hMSCs in a ratio of 300∶1 or left untreated (ctrl) for 3 days before Western blot analyses were carried out. Nuclear extracts were used to examine the phosphorylation status of Smad3 and CREB. Anti-ERK1/2 was used to control for equal loading.

Our data show that the activities of the cAMP and TGFβ signaling pathways are strongly dependent on culture conditions. The higher basal cAMP and Smad3 levels in 3D suspension cultures coincide with higher Smad3 activation and gene expression in response to TGFβ. We wondered whether environmental factors, such as stromal cells, may also affect the activities of the cAMP and TGFβ pathways. To test this hypothesis, we co-cultured bone marrow-derived mesenchymal stem cells (MSCs) [30], which have been shown to enter breast cancer lesions and interact with breast cancer cells [31], [32], with MDA-MB-231 cells. We found that as few as 1 MSC per 300 breast cancer cells was able to induce Smad3 and CREB phosphorylation in breast cancer cells (Fig. 11). This shows that environmental factors are able to simultaneously activate the cAMP and TGFβ signaling pathways which then could cooperate to regulate gene expression.

## Discussion

The data presented here suggest that cAMP promotes TGFβ/Smad3-mediated expression in breast cancer cells by upregulating the expression of the TGFβ receptor TβRI. This conclusion is based on a number of observations. (i) Half of the tested genes, group B genes, show responsiveness to forskolin only in the presence of TGFβ. (ii) Forskolin enhances TGFβ-mediated phosphorylation of TGFβ effector Smad3, without affecting Smad3 phosphorylation in the absence of TGFβ. (iii) Forskolin substantially upregulates the expression of TβRI. (iv) A constitutively active version of TβRI mimics the effect of cAMP on Smad3 phosphorylation and on TGFβ-mediated gene expression.

In 3D-cultured cells, forskolin failed to enhance TGFβ-dependent gene expression and Smad3 phosphorylation and to upregulate TβRI levels. Cells in 3D culture were different to 2D-cultured cells in many aspects of which some that are relevant to this study are listed in Figure S1B. Among these are the higher levels of TβRI RNA and protein, total Smad3 protein and basal cAMP. The reason for these differences could not be accounted to different oxygen supplies in 2D- vs. 3D-cultured cells, since expression of the hypoxia marker carbonic anhydrase IX [33] was comparable under the two culture conditions (Figure S1C). The higher level of total Smad3 in 3D-cultured cells may have alone been sufficient to prevent forskolin from enhancing TGFβ signaling, since overexpression of Smad3 abolished the forskolin effect on TGFβ signaling in 2D-cultured cells (Fig. 5D). Overexpression of Smad3 also increased TGFβ-dependent Smad3 phosphorylation and may therefore at least partially also be responsible for the higher phosphorylation status of Smad3 in TGFβ-treated 3D-cultured cells. The higher expression of the TβRI enzyme may have further increased Smad3 phosphorylation. Besides higher total Smad3 and TβRI expression, TβRI in 3D-cultured cells was also refractory to the stimulatory effect of forskolin. TβRI levels may have reached a certain threshold level above which a further rise in response to cAMP was not possible. Hence, both the high Smad3 level and the resistance of TβRI to forskolin may have contributed to the lack of the ability of forskolin to increase Smad3 phosphorylation and TGFβ-dependent gene expression in 3D-cultured cells.

Theoretically, the degree of Smad3 phosphorylation at Ser423 and S425 as monitored here by the anti-P-Smad3 antibody might not be entirely dependent on TβRI. However, so far, neither PKA, nor any other kinase has been described to be able to phosphorylate Smad3 at these positions [34]. Hence, it is very unlikely that cAMP induced Ser423/S425 phosphorylation directly via PKA instead of acting indirectly via TβRI. It might also be possible that cAMP stimulated a PKA/TβRI interaction. However, to our knowledge, such interaction has not yet been reported. In addition, the forskolin effect on TGFβ signaling was dependent on active transcription. Hence, it is likely that the higher phosphorylation of Smad3 on Ser423/S425 in forskolin-treated 2D-cultured cells was exclusively the result of a higher expression of TβRI. This hypothesis is supported by our finding that overexpression of TβRI was sufficient to increase Smad3 phosphorylation and TGFβ/Smad3-dependent gene expression (Fig. 8B).

Besides its effect on TβRI expression, cAMP could have also modulated the activities of other regulatory proteins that determine the fate of phosphorylated Smad3. Among those Smad3-regulating proteins is YAP. YAP regulates Smad3 nucleocytoplasmic shuttling in a cell density-depending manner [27]. At high cell density, YAP is phosphorylated and prevents phospho-Smad3 from entering the nucleus. In 3D-culture, cells showed higher phospho-YAP levels consistent with the notion that 3D-cultured cells are more tightly attached to each other. However, phospho-YAP levels did not seem to be coupled to nuclear Smad3 levels in MDA-MB-231 cells, since down-regulation of YAP expression had no significant effect on the abundance of phospho-Smad3 in the nucleus. Nevertheless, YAP interfered negatively with the forskolin effect on TGFβ-driven expression suggesting a Smad-independent effect of YAP on TGFβ signaling.

Another factor that regulates Smad3 activity is TRB3 (Tribbles homolog 3). TRB3 increases the stability of phospho-Smad3 by down-regulating Smurf2 (Smad ubiquitin regulatory factor 2) [35], an E3 ubiquitin ligase that triggers Smad3 degradation [36]. In addition, TRB3 promotes nuclear translocation of Smad3. Checking on TRB3 expression under the different conditions, we did neither find an increased TRB3 expression in the presence of forskolin, nor a higher level in 3D-cultured cells (data not shown). This rules out the possibility that TRB3 may have played a role in mediating the effect of forskolin on TGFβ-mediated expression and it also suggests that TRB3 is not responsible for the higher phospho-Smad3 level in TGFβ-treated 3D- vs. 2D-cultured cells.

Previous studies on cross-talk between the TGFβ and cAMP pathways were conducted on fibroblasts, endothelial and keratinocytes. Mostly, antagonistic interactions have been reported [26], [37]–[41]. In human dermal fibroblasts, cAMP was shown to counteract TGFβ/Smad3-dependent expression of PAI-1 and other genes by blocking the interaction of phospho-Smad3 with the transcriptional co-factor CBP (CREB binding protein)/p300 [42]. Also, in adrenocortical cells, cAMP suppressed Smad3 expression [43]. It seems, therefore, that breast cancer cells, epithelial cells and fibroblasts are using cAMP very differently to modulate TGFβ-dependent signaling. Interestingly, *vice versa*, TGFβ was shown to modulate the PKA/CREB signaling pathway in colon cancer by increasing PKA activity and CREB phosphorylation leading to increased apoptosis [44]. In MDA-MB-231 cells, however, CREB phosphorylation was not found to be changed in response to TGFβ (data not shown).

The analysis of the mechanism by which cAMP raises TβRI expression in MDA-MB-231 cells revealed that the cAMP effect requires transcription to be active. Promoter assays showed that cAMP is able to significantly, though moderately, activate a TβRI promoter fragment suggesting that cAMP exerts its effect on TβRI expression at least partially by directly affecting TβRI transcription. However, RNA interference experiments demonstrated that the major mediator of cAMP actions on transcription, CREB, was dispensable for the cAMP effect on TβRI. In line with this observation, no CREB binding site could be found within the TβRI promoter fragment analyzed (data not shown). The known TβRI-regulating transcription factor Six1 [19] did not seem to be involved in the cAMP effect either, since neither Six1 overexpression enhanced the ability of cAMP to activate TβRI promoter activity, nor did forskolin increase Six1 expression. We also analyzed the responsiveness of Six1 co-factor Eya2, which also plays a role in Six1-mediated TβRI expression [45], to cAMP. It was found that forskolin is unable to substantially increase the expression level of Eya2 (data not shown). Interestingly, inhibition of histone deacetylases increased TβRI expression in MDA-MB-231 cells, an effect also observed with MCF-7 and ZR75 breast cancer cells [46]. The extent by which HDACi upregulated TβRI expression was comparable to that induced by forskolin. Simultaneous treatment of forskolin and HDACi it did not increase TβRI expression suggesting that cAMP acted on TβRI expression by increasing histone acetylation.

Along with the cAMP-enhanced TGFβ responses we found decreased cellular proliferation. The strongest negative effect on proliferation was seen when both forskolin and TGFβ1 were added. These effects may be mediated by the cell cycle inhibitor p21 whose TGFβ-driven expression was enhanced by forskolin. p21 has been shown to induce cell cycle arrest in MDA-MB-231 and other breast cancer cells [47]. Recently, in addition to its anti-proliferative activity, a novel tumor-promoting effect of p21 on MDA-MB-231 and other breast cancer cells have been described leading to enhanced cell migration and invasion [48]. The combined effect of cAMP and TGFβ on PAI-1, Cox-2, and PTHrP suggests additional effects on breast cancer function. PTHrP has been shown to be involved in bone metastasis [20] as well as in tumor initiation and progression [49]. Cox-2 is involved in metastasis formation by MDA-MB-231 cells [50], [51] and PAI-1 expression is associated with unfavorable prognosis [52].

Besides the effects on these specific genes, the TGFβ/cAMP interaction may be of more general importance. TGFβ is known to have dual activities on cancer progression, one as a tumor suppressor and the other as tumor promoter [53]. The tumor-promoting effect is predominantly the result of TGFβ’s ability to induce epithelial-to-mesenchymal transition (EMT) leading to a more invasive cell phenotype with stem-cell characteristics [54], [55]. For the induction of EMT by TGFβ, TβRI and Smad3 are major players [19], [53]. Apparently, EMT induced by TGFβ is accompanied by prolonged increased activity of Smad3 [53], just as was found here in the presence of forskolin. Hence, a rise in the cAMP level may help to direct the TGFβ effect towards EMT. EMT is a major inducer of cancer stem cells (CSCs) [56] which play a crucial role in tumor initiation, progression and metastasis in breast cancer [57]. Interestingly, overexpression of Six1, a major activator of TβRI transcription in breast cancer [19], increased the CSC pool in breast cancer cells by activating the TGFβ pathway [58]. Since cAMP also enhances TGFβ signaling through TβRI, it may as well support TGFβ’s ability to generate CSCs. Breast CSCs themselves show a high activity of the TGFβ pathway [59], [60] suggesting that the TGFβ pathway is also important for CSC maintenance. Again, cAMP could support the function of TGFβ. The CSC pool can also be increased by mesenchymal stem cells (MSCs) [61]. Here, we show that MSCs are able to increase both Smad3 and CREB phosphorylation in breast cancer cells suggesting that MSCs are able to induce the TGFβ and cAMP pathway at the same time allowing these pathways to communicate. It is possible that this dual activation plays a role in the stimulatory effect of MSCs on the CSC pool. Besides its effect on CSCs, TGFβ may particularly affect triple-negative breast cancers, a subtype devoid of estrogen receptor, progesterone receptor and Her2. Triple-negative breast cancers, to which MDA-MB-231 cells belong, show higher metastatic potential, when the TGFβ/Smad3 pathway is more active [62]. Hence, cAMP may as well support the metastatic activity of TGFβ on triple-negative breast cancers. Another report shows that a certain genotype of TGFβ1 is associated with an increased risk to develop a progesterone receptor-negative breast cancer [63]. Again, cAMP may promote this effect.

In conclusion, by supporting TGFβ/Smad3 signaling cAMP may enhance TGFβ/Smad3-induced EMT, generation of CSCs and breast cancer metastasis, thereby, deteriorate the outcome of breast cancer patients. In support of this notion, higher cAMP levels, as reflected by higher CREB phosphorylation, is associated with a poor prognosis [12].

## Supporting Information

Figure S1 **MDA-MB-231 cells in 2D and 3D cultures are distinct in many features.** (A) Micrographs of 2D- and 3D-cultured MDA-MB-231 cells. (B) Differences between 2D- and 3D-cultured MDA-MB-231 cells. (C) Hypoxia marker carbonic anhydrase IX (CAIX) was similarly expressed in 2D- and 3D-cultured MDA-MB-231 cells suggesting that the oxygen supply to the cells was similar under both culture conditions. After incubation of cells in 2D or 3D culture, plasma membrane extracts were analyzed for CAIX levels by Western blot analysis. Coomassie stain serves as a protein loading control.
(TIF)

Figure S2 **In MDA-MB-231 cells, phospho-YAP is exclusively found in the cytoplasm, whereas phospho-Smad3 is present in the nucleus.** Western blot analysis of cytoplasmic extracts (CE) and nuclear extracts (NE) from 3D-cultured cells for P-YAP and P-Smad3 in the presence of TGFβ. For comparison, also banding pattern for GAPDH and Coomassie-stained proteins are shown.
(TIF)
