# Supplementary figures and images for: Cyclic AMP Enhances TGFβ Responses of Breast Cancer Cells by Upregulating TGFβ Receptor I Expression
Source: PLoS One. 2013 Jan 18;8(1):e54261. doi: 10.1371/journal.pone.0054261 (PMC3548810; doi:10.1371/journal.pone.0054261)

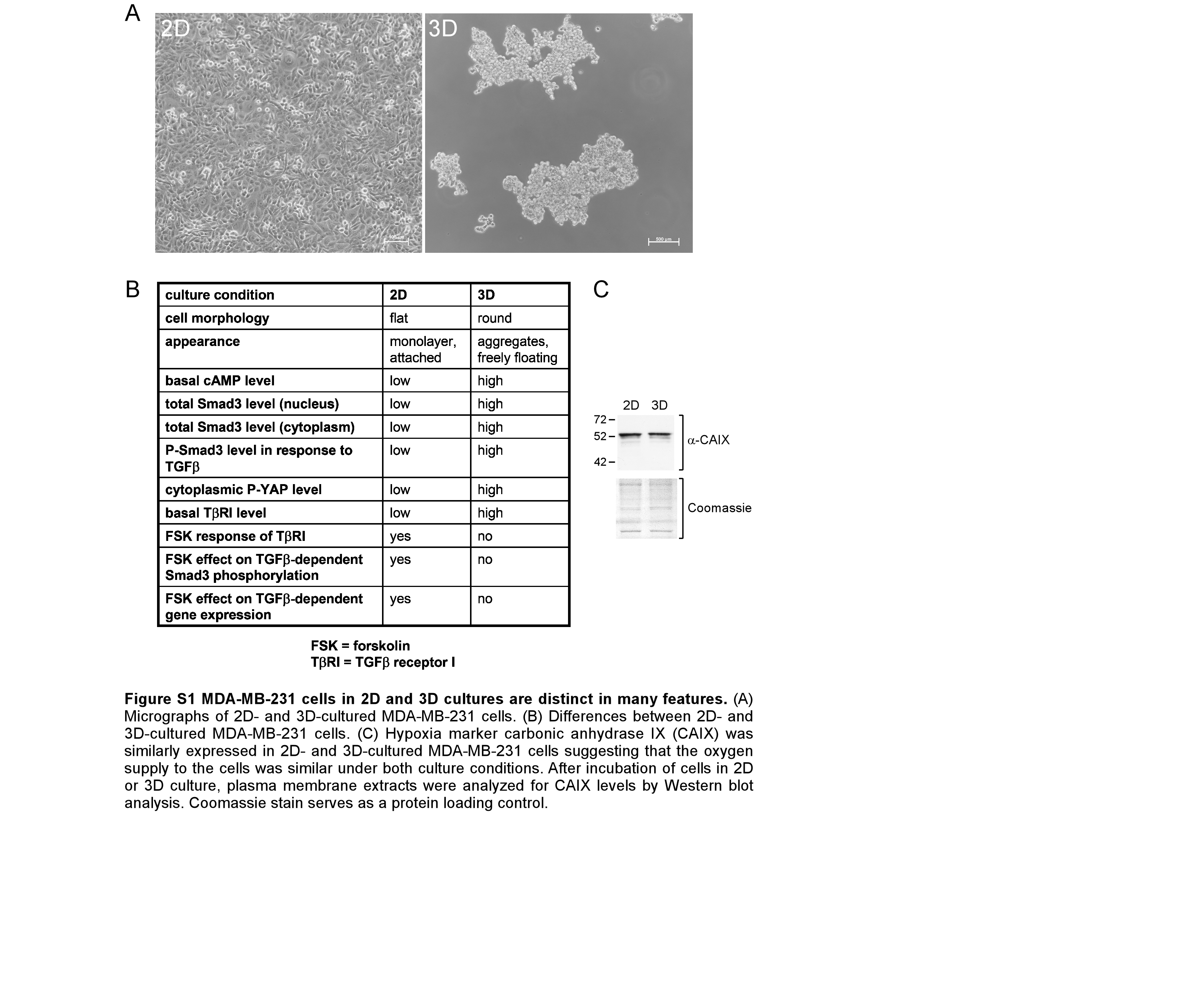

Supplement: Figure S1 — MDA-MB-231 cells in 2D and 3D cultures are distinct in many features. (A) Micrographs of 2D- and 3D-cultured MDA-MB-231 cells. (B) Differences between 2D- and 3D-cultured MDA-MB-231 cells. (C) Hypoxia marker carbonic anhydrase IX (CAIX) was similarly expressed in 2D- and 3D-cultured MDA-MB-231 cells suggesting that the oxygen supply to the cells was similar under both culture conditions. After incubation of cells in 2D or 3D culture, plasma membrane extracts were analyzed for CAIX levels by Western blot analysis. Coomassie stain serves as a protein loading control. (TIF) [file pone.0054261.s001.tif]

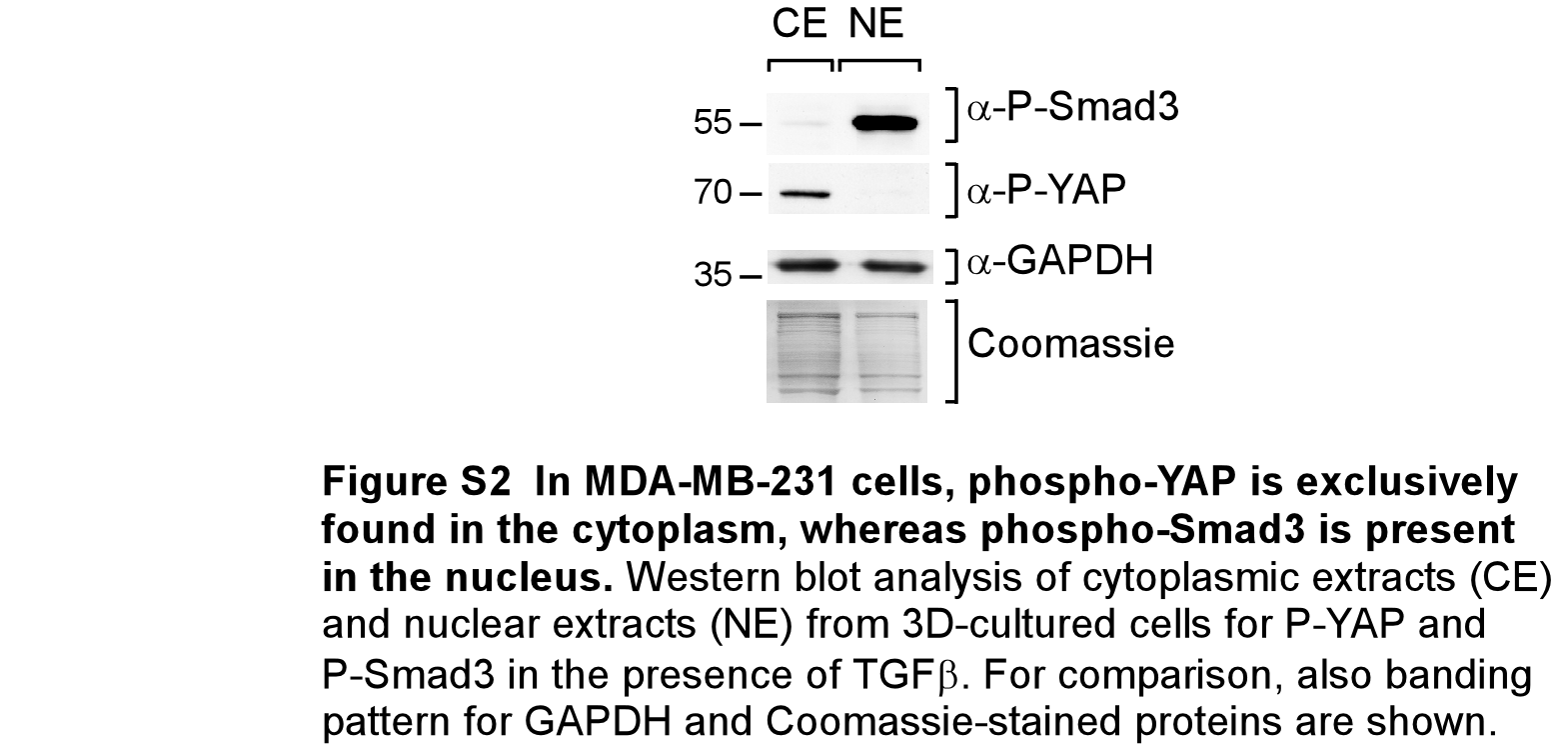

Supplement: Figure S2 — In MDA-MB-231 cells, phospho-YAP is exclusively found in the cytoplasm, whereas phospho-Smad3 is present in the nucleus. Western blot analysis of cytoplasmic extracts (CE) and nuclear extracts (NE) from 3D-cultured cells for P-YAP and P-Smad3 in the presence of TGFβ. For comparison, also banding pattern for GAPDH and Coomassie-stained proteins are shown. (TIF) [file pone.0054261.s002.tif]
